# Presence of Lactic Acid Bacteria in the Intestinal Tract of the Mediterranean Trout (*Salmo macrostigma*) in Its Natural Environment

**DOI:** 10.3390/life11070667

**Published:** 2021-07-07

**Authors:** Massimo Iorizzo, Gianluca Albanese, Bruno Testa, Mario Ianiro, Francesco Letizia, Mariantonietta Succi, Patrizio Tremonte, Mariasilvia D’Andrea, Nicolaia Iaffaldano, Raffaele Coppola

**Affiliations:** Department of Agriculture, Environmental and Food Sciences, University of Molise, Via De Sanctis, 86100 Campobasso, Italy; iorizzo@unimol.it (M.I.); g.albanese@studenti.unimol.it (G.A.); m.ianiro@studenti.unimol.it (M.I.); f.letizia@studenti.unimol.it (F.L.); succi@unimol.it (M.S.); tremonte@unimol.it (P.T.); dandrea@unimol.it (M.D.); nicolaia@unimol.it (N.I.); coppola@unimol.it (R.C.)

**Keywords:** lactic acid bacteria, gut microbiota, Mediterranean trout, river environment

## Abstract

Knowledge of the composition of the gut microbiota in freshwater fish living in their natural habitat has taxonomic and ecological importance. Few reports have been produced on the composition of the gut microbiota and on the presence of LAB in the intestines of freshwater fish that inhabit river environments. In this study, we investigated the LAB community that was present in the gastrointestinal tract (GIT) of Mediterranean trout (*Salmo macrostigma*) that colonized the Biferno and Volturno rivers of the Molise region (Italy). The partial 16S rRNA gene sequences of these strains were determined for the species-level taxonomic placement. The phylogenetic analysis revealed that the isolated LABs belonged to seven genera (*Carnobacterium, Enterococcus, Lactobacillus, Lactiplantibacillus, Vagococcus, Lactococcus,* and *Weissella*). The study of the enzymatic activities showed that these LABs could contribute to the breakdown of polysaccharides, proteins, and lipids. In future studies, a greater understanding of how the LABs act against pathogens and trigger the fish immune response may provide practical means to engineer the indigenous fish microbiome and enhance disease control and fish health.

## 1. Introduction

The intestinal tracts of fishes contain a complex and dynamic community of microorganisms [1,2]. Among them, the lactic acid bacteria (LAB) play an important role due to some of their properties: production of antimicrobial substances and improved disease resistance, greater antioxidative stress tolerance, immune response stimulation, and increase in the availability of nutrients [3,4,5]. LABs were among the first living organisms on Earth; they appeared about three billion years ago in the transition period from anaerobiosis to aerobiosis. They seem to have adapted well to both anaerobic and aerobic life conditions since they bear all the necessary proteins for respiration and several enzymes involved in fermentative pathways [6]. Several species of LABs belonging to the *Lactobacillus, Lactococcus, Leuconostoc, Enterococcus, Streptococcus, Carnobacterium, Pediococcus*, and *Weissella* genera have adapted to grow under very different environmental conditions and are important representatives of the gut microbiota of both freshwater and marine fish [7]. In recent decades, numerous studies have shown that different intrinsic and extrinsic factors affect the level, composition, and/or diversity of the fish gut microbiota: life stage, trophic level, diet, seasonality, habitat, stress, sex, and phylogeny [8,9,10]. LAB’s diversity was particularly studied in freshwater fish, but few reports have been produced on the composition of the gut microbiota and on the presence of LABs in the intestines of freshwater fish that inhabit river environments [11,12,13,14].

**Figure 1:**
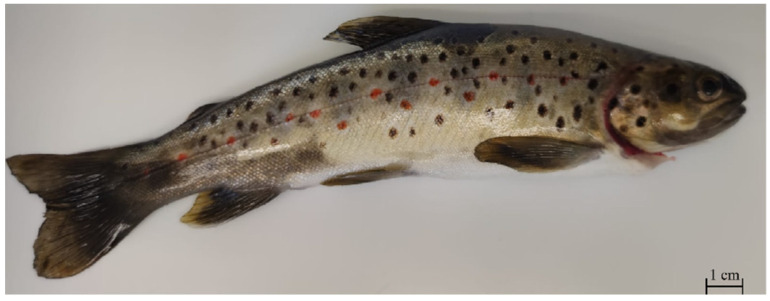
Adult specimen of Mediterranean trout.

Knowledge of the composition of the gut microbiota in freshwater fish living in their natural habitat has taxonomic and ecological importance. In particular, the study of the gut microbiome in threatened wildlife species has enormous potential to improve conservation efforts and to gain insights into the host–microbe coevolution. The interaction of animals with their respective symbiotic microorganisms can provide important tools for the management of various issues related to the protection of endangered animal species [15]. *Salmo macrostigma* is an endemic freshwater trout species of the Mediterranean area (Figure 1). This salmonid is protected by the Habitats Directive 92/43/EEC and is considered a “vulnerable species” in Europe and “critically endangered” in Italy [16]. The diet of the Mediterranean trout consists mainly of macrozoobenthos, larvae and adult insects, and to a lesser extent, vegetable elements, crustaceans, and mollusks. Macroinvertebrates and fry and small fish, as well as being food competitors, also represent a valid source of nourishment in the vital stages of the Mediterranean trout. The water temperature in this species’ thermal niches is between 7 and 15 °C, which is the optimal survival range for this cold stenothermal fish species [17,18,19,20]. In this study, we investigated the LAB community present in the gastrointestinal tract (GIT) of the Mediterranean trout (*Salmo macrostigma*) that inhabits river environments. At present no similar studies are available on this freshwater fish species.

## 2. Materials and Methods

### 2.1. Lactic Acid Bacteria (LAB) Isolation

**Figure 2:**
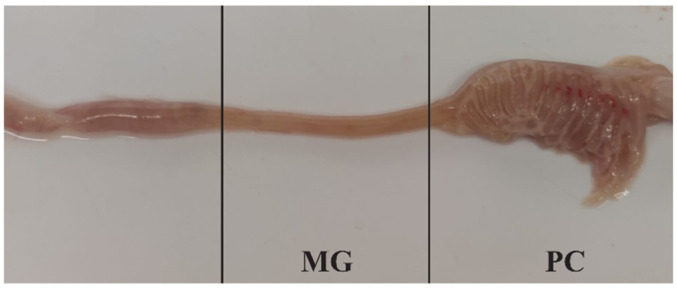
The intestinal tract of an adult Mediterranean trout. Pyloric ceca (PC) and midgut (MG) sections.

A total of 18 adult specimens of Mediterranean trout that accidentally died in the fish traps used in the scientific activities of the LIFE Nat. Sal. Mo Project (LIFE17 NAT/IT/000547) was collected. The fish were taken from 7 different study sites on the Biferno (4 sites) and Volturno (3 sites) rivers (Molise region, Italy) between November and January during the daily inspection of the fish traps and immediately transported in portable refrigerators (2–8 °C) to the microbiology laboratory. The exterior of the fish was wiped clean with 70% ethanol, the abdomen opened at the ventral midline, and the whole intestine was aseptically removed from the abdominal cavity and separated into the proximal (pyloric ceca, PC) and midgut (MG) sections (Figure 2).

Once the specimens were longitudinally opened, the MG sections were rinsed using 3% NaCl to remove non-adherent (allochthonous) bacteria and digesta. Later, the PC and MG matrices were homogenized together in sterile physiological water (0.9% NaCl) and serial decimal dilutions were obtained. The bacterial colonies were isolated by plating serial decimal dilutions on MRS and M17 media (Oxoid, Milan, Italy) that were supplemented with cycloheximide (40 mg/L). Plates were incubated at 30 °C under anaerobic conditions using an anaerobic system (AnaeroGen, Oxoid, Milan, Italy). After 48–72 h, approximately 5% of the colonies were isolated and purified from culture plates by streaking them on the respective culture media. The bacterial colonies were randomly selected according to morphological differences (colony size and shape).

### 2.2. Phenotypic and Biochemical Characterization

Prior to genotypic identification, presumptive LABs isolates were examined for their Gram reactions and catalase activity. The Gram reactions were performed by dissolving a loop full of freshly grown colony material in a drop of 3% KOH (Sigma-Aldrich; St. Louis, MO, USA) on a microscope slide. Like the Gram stain reaction, the KOH test is based on differences in the chemistry of the bacterial cell wall. In the presence of potassium hydroxide, Gram-negative cell walls are broken down. KOH easily dissolves the thin layer of peptidoglycan of the cell walls of Gram-negative bacteria. On the other hand, Gram-positive bacteria are not affected by KOH because they have a thicker peptidoglycan layer in the cell wall. The isolates, which did not give a viscid product, were selected since LAB are known as Gram-positive cells [21].

The catalase activity was determined by adding a drop of hydrogen peroxide (H_2_O_2_; Sigma-Aldrich) solution (5%) to a small quantity of colony mass on a glass slide. The catalase test differentiates bacteria that produce a catalase enzyme that can degrade H_2_O_2_ in water and free oxygen. The production of oxygen bubbles can be observed.

After genotypic identification, the enzyme activity of the LABs was determined using an API-ZYM system (bioMérieux SA, Marcy l’Etoile, France) according to the manufacturer’s instructions.

### 2.3. Genotypic Identification

Genomic DNA from pure cultures of putative LABs was extracted using a Bacterial Genomic DNA Isolation Kit (Norgen Biotek, Thorold, ON, Canada) according to the manufacturer’s instructions. The 16S rRNA gene fragments were amplified using the 27F and 1492R primer pair [22].

The polymerase chain reaction (PCR) mixture contained 10 μL 2× PCR master mix (Norgen Biotek), 0.5 μL of each primer (2.5 μM), 7 μL Milli-Q water, and 2 μL template DNA. In the negative control for the PCR reactions, Milli-Q water was used instead of DNA. The PCR reactions were performed with a Mastercycler Nexus PCR thermal cycler (Eppendorf, Hamburg, Germany). PCR amplifications were achieved using the following program: pre-denaturation at 95 °C for 10 min, then 30 cycles of denaturation at 95 °C for 30 s, annealing for 1 min, and extension at 72 °C for 1.5 min. The last cycle was followed by a 7 min extension at 72 °C [23]. PCR products were analyzed using electrophoresis on a 1.0% (*w*/*v*) agarose gel in a 1× Tris-Borate-EDTA (TBE) buffer.

The bands were visualized under a UV transilluminator (Bio-Rad, Hercules, CA, USA) and the sizes were estimated by comparison against 1 kb DNA ladder (Norgen Biotek).

PCR products were purified using a QIAquick PCR purification kit (QIAGEN GmbH, Hilden, Germany) and sent to a commercial facility for sequencing (Eurofins MWG Biotech Company, Ebersberg, Germany). The 16S rRNA sequences were examined using the Basic Local Alignment Search Tool (BLAST) [24] program and were compared with known reference databases in the National Center for Biotechnology Information (NCBI) for taxonomic placement [25].

## 3. Results

### 3.1. LAB Species Diversity

In total, sixty-one Gram-positive and catalase-negative bacterial strains were presumptively considered LABs. The partial 16S rRNA gene sequences of these strains were determined for taxonomic placement. The BLASTN algorithm was applied to the GenBank database to identify sequences (http://www.ncbi.nlm.nih.gov/BLAST/ (accessed on 26 June 2021). The sequence of the closest related type strain was compared with the respective sequence of our collected strains.

Sequence matches that showed high identity scores (98% and above) were considered acceptable for taxonomic placement at the species level [26].

**Figure 3:**
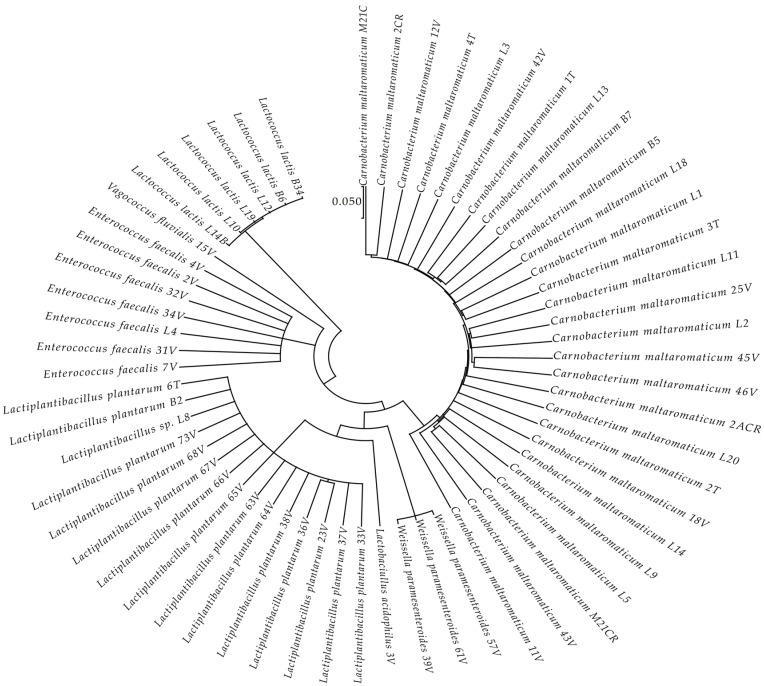
Results of the phylogenetic analysis of 16S rRNA gene sequences of the 61 LABs isolates compared with the sequences of type strains from the National Centre for Biotechnology Information (NCBI). The analysis was conducted with the MEGA X program [27] using the maximum likelihood method and the Hasegawa–Kishino–Yano model [28]. The scale bar represents a 5% nucleotide sequence difference.

According to the 16S rRNA gene sequences, all isolated strains and their related type strains were used to construct a phylogenetic tree (Figure 3) using the MEGA X program [27] via the maximum likelihood method and the Hasegawa–Kishino–Yano model [28]. The partial 16S rRNA gene sequences obtained during the identification of LAB isolates were submitted to the GenBank database. Table S1 (Supplementary Material) shows the list of isolates LABs with the corresponding GenBank accession numbers and the taxonomic references.

**Figure 4:**
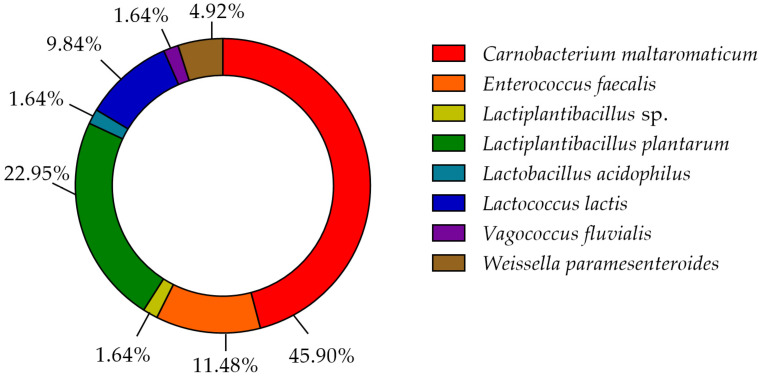
Donut chart showing the percentage distribution of the LAB species (61 strains) that were isolated from the intestinal tracts of the Mediterranean trout.

The phylogenetic analysis revealed that the 61 strains belonged to 7 genera (*Carnobacterium*, *Enterococcus*, *Lactobacillus*, *Lactiplantibacillus*, *Vagococcus*, *Lactococcus*, and *Weissella*). The majority of the LAB isolates were identified as *Carnobacterium maltoaromaticum* (28 strains), *Lactiplantibacillus plantarum* (14 strains), *Enterococcus faecalis* (7 strains), *Lactococcus lactis* (6 strains), and *Weisella paramesenteroides* (3 strains). A minority of LABs were identified as *Lactobacillus acidophilus* and *Vagococcus fluvialis* (1 strain each). It was not possible to determine whether the L8 strain belonged to the species *Lp. plantarum* or *Lp. pensosus*. Our results confirmed that the definition of the phylogenetic distances and sometimes even the differentiation using 16S RNA sequencing was not feasible for *Lp. plantarum* and *Lp. pentosus* because of the high similarity. In fact, these two species belong to the same phylogenetic group (*Lp. plantarum* group) [29,30]. In Figure 4, the percentage distribution of LABs species is shown. *C. maltoaromaticum* was widely found in 12 trout specimens (percentage frequency 66.67%), *Lp. plantarum* was detected in 7 trout specimens (44.44%), while *Lc. lactis* and *E. faecalis* were detected in 27.78% of the trout specimens. Table 1 shows the presence and frequency (%) of every LAB species in the intestinal tract of the 18 trout specimens.

### 3.2. Biochemical Characterization

The enzymatic activities assayed using the API-ZYM system are presented in Table 2. All LAB isolates showed no activities for the enzymes β-glucuronidase, α-mannosidase, and trypsin. The acid phosphatase, lipase, and α-chymotrypsin enzymes were not detected in the *Lp. plantarum* strains, which instead exhibited the following enzymatic activities: alkaline phosphatase, β-galactosidase, α-glucosidase, β-glucosidase, and N-acetyl-β-glucosaminidase. All the strains belonging to *C. maltaromaticum* species showed acid phosphatase, cystine arylamidase, leucine arylamidase, valine arylamidase, naphthol-AS-BI-phosphohydrolase, and N-acetyl-β-glucosaminidase activities. Meanwhile, α-mannosidase, α-fucosidase, α-chymotrypsin, and lipase activities were not detected in *C. maltaromaticum* species. Lipase and α-chymotrypsin activities were detected only in *Lc. lactis* strains.

In the group of bacteria ascribed to the *C. maltaromaticum*, *Lp.plantarum*, and *Lc. Lactis* species, there was variability in some enzymatic activities; the positive strains percentage is highlighted in Table 2 using sparkline charts.

## 4. Discussion

Our results highlight that in the LAB community of the gut microbiota of the Mediterranean trout, there is a predominance of *C. maltaromaticum* and *Lp. plantarum*. There are no similar studies available on this freshwater fish for comparison. Diet and environmental temperature are among the factors that have the greatest impact on the gut bacterial communities of fishes [8,9,10,31,32]. Therefore, it is assumed that the protein-rich diet of the Mediterranean trout and the temperature of the water (5–15 °C) in which it lives also affects the composition of the intestinal LAB community [33]. The results obtained by Bucio et al. [13] in a study on the gut microbiota of 11 other freshwater fish species taken from rivers and fish farms highlighted the minor presence of *Lp. plantarum* and the absence of *C. maltaromaticum*. Instead, other studies showed that *C. maltaromaticum* are important inhabitants of the gastrointestinal tract of freshwater and marine fishes [3,11,12,34,35,36,37,38]. This bacterium is capable of growing in harsh conditions, such as low temperature, low pressure, and anoxic conditions; moreover, it was found that the temperate/polar aquatic and terrestrial environments are both natural habitats [39].

Although a fish probiotic culture was used [40,41], several cases of disease associated with *C. maltaromaticum* were reported [42,43,44]. However, a recent study showed that the bacterial virulence factor was present only in some strains derived from diseased fishes [45]. *Lp. plantarum* is a versatile and ubiquitous microorganism that is capable of colonizing several ecological niches, including the gastrointestinal tract of mammals, insects, and fishes [3,46,47]. Our results have shown that it is a predominant species in the community of LABs that populate the gut microbiota of *S. macrostigma*. Other studies have shown the presence of *Lp. plantarum* in the intestinal tract of other freshwater fish [48] and some strains are used as probiotics in aquaculture practices [5,49,50]. In our study, the *Lc. lactis* species was isolated amongst the LAB in the Mediterranean trout intestine, which is in agreement with other studies carried out on salmonids [3,51,52]. It has generally been reported that *Lc. lactis* strains are highly adaptable to different environments, including animal sources, dairy products, and silages [53]. Some researchers have hypothesized that because *Lc. lactis* is a LAB found in milk and milk derivatives, it may also be present in the disposal effluents of dairy factories that are released into the environment [54]. Nevertheless, its frequent isolation from the intestines of freshwater and marine fish in areas that are not close to dairy industries has caused this hypothesis to be abandoned [55,56,57]. Furthermore, the phenotypic diversity between *Lc. lactis* strains derived from fish intestines and *Lc. lactis* strains derived from milk derivatives were demonstrated [58,59]. Recently, some strains of *Lc. lactis* were selected and proposed as probiotics in the fish diet [35,60].

The *Enterococcus* genus includes species that predominantly reside in the gastrointestinal tract of humans and animals; nonetheless, they are widely distributed in the environment [61,62]. The results of our survey have highlighted the presence of *E. faecalis* in the intestinal tract of *S. macrostigma*, confirming its frequent presence in the gut microbiota of fishes [3,63]. The *E. faecalis* are resilient and versatile species that are able to survive under harsh conditions [64]. Some strains of this species have been proposed as probiotics in aquaculture [5,65].

Our results confirmed that, more or less frequently, some LABs belonging to the *V. fluvialis*, *W*. *pseudomesenteroides*, and *L. acidophilus* species can be isolated from fish guts [3,12,51,66,67].

Bacteria present in the aquatic environment and introduced with food may influence the composition of the gut microbiota in fish [68].

LABs are ubiquitous microorganisms and several studies found the presence of species such as *C. maltoaromaticum* and *Lp. plantarum* in the aquatic environment [69,70] and in the microbiota of insects [47,71]. The reasons as to why the presence of these LAB species are recurrent in the intestinal tracts of several fish species and the reasons for the mutual relationships with the host needs to be understood.

Our study was conducted using a culture-dependent method; further studies using culture-independent methods will be applied in the future (e.g., next-generation sequencing (NGS)) for the assessment of biodiversity in gut microbiota communities of wild Mediterranean trout. However, the technique we adopted allowed us to isolate the most numerically representative LABs from the intestinal tract of this salmonid and to submit them to a first characterization based on the in vitro evaluation of their enzymatic profile.

The environmental temperature and the host trophic levels (herbivorous, carnivorous, omnivorous) influenced the composition and metabolic capacity of the gut microbiota of wild freshwater fishes [33,72,73,74]. The gut microbiota plays a major role in the nutrition, growth, health, and survival of the host fish [75]. Specific bacteria composing the gut microbiota are involved in the breakdown of large food molecules (i.e., polysaccharides, proteins, fats, nucleic acids) [76].

The LABs are producers of extracellular enzymes that are involved in the breakdown of cellulose, starch, proteins, and lipids [77,78,79,80]. In our research, all *C. maltoaromaticum* strains displayed aminopeptidase activity (leucine, cystine, and valine arylamidase) as proteolytic indicators and the alkaline and acid phosphatase activities involved in lipidic metabolism [81,82].

All the *C. maltoaromaticum* and *Lp. plantarum* strains showed N-acetyl-β-glucosaminidase activity. LABs producing this enzyme could facilitate the breakdown of the exoskeleton of many invertebrates, including insects, which are the prey of the Mediterranean trout in its natural habitat [83]. The exoskeletons consist mainly of chitin, a linear polysaccharide composed of N-acetylglucosamine subunits linked via β-1,4 bonds [84].

The lipase and α-chymotrypsin activities were detected only in *Lc. lactis* strains. These data confirm the potential of this bacterial species in the breakdown of proteins and lipids [85]. In the group of bacteria ascribed to species *C. maltaromaticum*, *Lp. plantarum*, and *Lc. lactis*, there was variability in some enzymatic activities. However, the limited number of isolated LAB and the techniques used did not allow us to carry out a meaningful analysis on the intraspecific variability (phenotypic and genotypic). In the future, when our collection of bacteria is numerically more substantial, further studies on these important aspects will be carried out.

The characterization of microbial populations in the intestinal microenvironment of fish and understanding the physiological interactions between the indigenous microbiota and the host might have important implications.

However, the enzymatic properties of all the isolate strains shown in vitro did not axiomatically result in a positive contribution in digestive processes; this aspect deserves further investigation.

In contrast to endothermic animals, the exact role of gut microbiota in fish nutrition is difficult to conclude as a consequence of the complex and variable ecology of the GI tract of fish [86].

Moreover, among the microbial population in the fish gut, beneficial enzyme-producing bacteria continuously compete with pathogens through competitive exclusion; thus, this topic should be addressed in studies conducted in vitro and in vivo.

In general, the microbial population of the gut represents a very important and diversified enzymatic potential, and the enzymatic mass present in the digestive tract might interfere in a considerable way with a major part of the metabolism of the host animal [4,72].

Furthermore, the role of enzyme-producing fish gut bacteria as probiotics in the enhancement of food digestibility and their effect on gut enzyme activity was evaluated through several investigations [87,88].

Some studies have shown that the formation of the microbiota of the digestive tract from the larval stage to adult fish is formed gradually [89,90,91,92].

The enzyme-producing microorganisms isolated from fish digestive tracts can be beneficially used as a probiotic, especially in the larval stages [78]. The main strategy for using probiotics is to isolate intestinal bacteria with favorable properties from mature animals and include a large quantity of bacteria in the feed of immature animals of the same species [87,93,94].

In this context, the search for beneficial extracellular enzyme-producing gut bacteria to be used as probiotics for the fish may be of interest. Most studies on enzyme-producing gut bacteria isolated from fish were conducted on different fish species, while few studies have been carried out on salmonids [77].

Therefore, we believe that the topic enzyme-producing LABs isolated from fish deserves further investigations, especially in relation to chitinase activity, as chitin is one of the most renewable biopolymers on earth and might be useful as a constitutive material in formulated fish feed in the future. Even though dietary chitin modulates the intestinal microbiota and influences disease resistance, susceptibility, and innate immune parameters, these topics are not fully understood; as a consequence, further studies are needed [95].

The environmental temperature and the host diet create strong selective pressure in the gut, which shapes the structure of the gut microbial community [9,33,73,96]. *S. macrostigma* is a cold stenothermal fish species, which, in the natural environment, has a diet that is almost exclusively carnivorous. Our results suggest that these factors are important in shaping the gut microbial community of these fish. The microbial communities that constitute fish microbiomes are essential for the host’s health. Therefore, a better understanding of the natural bacterial communities of healthy individuals and how they interact with the host and other environmental factors is of crucial importance [97]. Captive animals tend to have different gut microbial communities compared with their wild counterparts [15,54,98]. The host–gut microbe mutualism evolved in a natural environment with complex climate patterns and food availability. A complete examination of host–intestine microbe dynamics must consider these factors [1]. This information would lay the foundations for exploring the impact of the gut microbiota composition and its function on the ecology, fitness, and evolution of their respective hosts. Discovering the core gut microbiome is crucial for understanding the ecology of microbial consortia and it is the first step toward defining a stable and healthy bacterial community in fish intestines. We believe that implementing the host–microbiota evolutionary process and microbial ecology into conservation policies would not only improve the efficiency of stocking programs for *S. macrostigma* but also for every fish species suffering a demographic decline [99].

The results obtained in this study show that the dominant LABs that are associated with the intestinal tract of the Mediterranean trout specimens examined belong to *C. maltoaromaticum* and *Lp. plantarum* species.

Our study is certainly not exhaustive and further genotypic and phenotypic investigations are underway on the gut microbiota of Mediterranean trout. These additional studies are needed to produce a greater understanding of the interactions between environment–host–microbiota and how specific microbes, such as LABs, can be used as resources to improve the health and wellbeing of this fish [81,82].

## Figures and Tables

**Table 1:** Occurrence and frequency of LAB species in the digestive tract of Mediterranean trout specimens.

LAB Species	Number of Strain Isolates	Host Trout Specimens	Total Trout Specimens	Frequency (%)
*Carnobacterium maltaromaticum*	28	12	18	66.7
*Lactiplantibacillus plantarum*	14	8	18	44.4
*Lactococcus lactis*	6	5	18	27.8
*Enterococcus faecalis*	7	4	18	27.8
*Lactobacillus acidophilus*	1	1	18	5.5
*Lactiplantibacillus* sp.	1	1	18	5.5
*Vagococcus fluvialis*	1	1	18	5.5
*Weisella paramesenteroides*	3	1	18	5.5

**Table 2:** Enzymatic profiles of 61 isolated LAB strains using the API-ZYM system (● positive, ● negative, ◐ variable). The number of isolated strains belonging to the different species is shown in parentheses, and for each enzymatic activity, the sparkline chart indicates the percentage of positive strains.

Enzyme Assayed	*C. maltoaromaticum*	*E. faecalis*	*Lactiplantibacillus* sp.	*Lp. plantarum*	*L. acidophilus*	*Lc. lactis*	*V. fluvialis*	*W. paramesenteroides*	Sparkline Chart (%)
	(28)	(7)	(1)	(14)	(1)	(6)	(1)	(3)	
Alkaline phosphatase	●	●	●	●	●	●	●	●	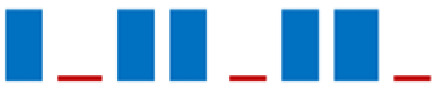
Esterase (C4)	●	●	●	◐	●	●	●	●	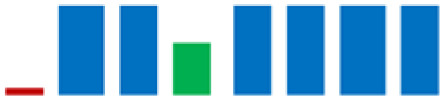
Esterase lipase (C8)	●	●	●	◐	●	●	●	●	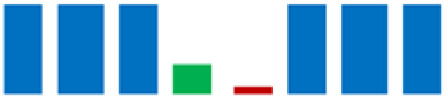
Lipase (C14)	●	●	●	●	●	◐	●	●	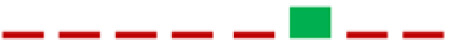
Leucine arylamidase	●	●	●	◐	●	●	●	●	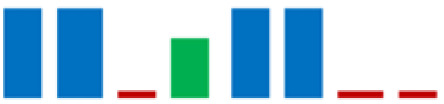
Valine arylamidase	●	●	●	◐	●	●	●	●	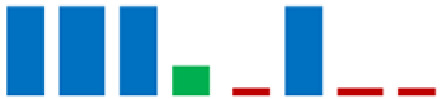
Cystine arylamidase	●	●	●	●	●	●	●	●	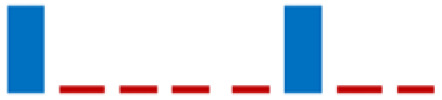
Trypsin	●	●	●	●	●	●	●	●	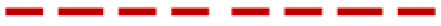
α-chymotrypsin	●	●	●	●	●	◐	●	●	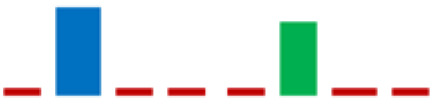
Acid phosphatase	●	●	●	●	●	●	●	●	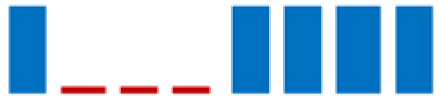
Naphthol-AS-BI-phosphohydrolase	●	●	●	◐	●	●	●	●	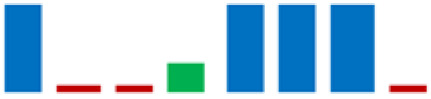
α-galactosidase	◐	●	●	◐	●	◐	●	●	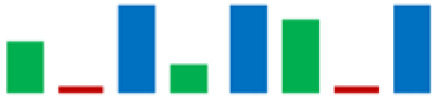
β-galactosidase	◐	●	●	●	●	◐	●	●	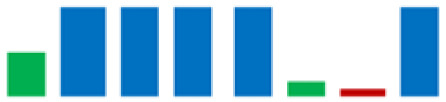
β-glucuronidase	●	●	●	●	●	●	●	●	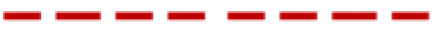
α-glucosidase	◐	●	●	●	●	◐	●	●	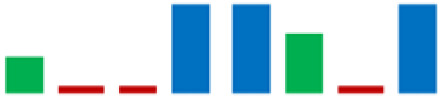
β-glucosidase	◐	●	●	●	●	◐	●	●	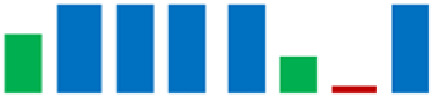
N-acetil-β-glucosaminidase	●	●	●	●	●	◐	●	●	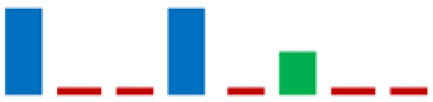
α-mannosidase	●	●	●	●	●	●	●	●	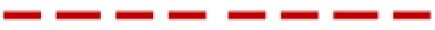
α-fucosidase	◐	●	●	◐	●	●	●	●	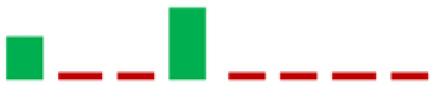

## Supplementary Materials

The following are available online at https://www.mdpi.com/article/10.3390/life11070667/s1. Table S1. List of the isolates LABs with corresponding GenBank (NCBI) accession numbers and the taxonomic references.
