# Supplementary material for: Presence of Lactic Acid Bacteria in the Intestinal Tract of the Mediterranean Trout (Salmo macrostigma) in Its Natural Environment
Source: Life (Basel). 2021 Jul 7;11(7):667. doi: 10.3390/life11070667 (PMC8306010; doi:10.3390/life11070667)
Supplement: Supplementary file 1 [file life-11-00667-s001.zip › life-1218403-supplementary.pdf]

life

Article

# Presence of Lactic Acid Bacteria in the Intestinal Tract of the Mediterranean Trout (*Salmo macrostigma*) in Its Natural Environment

**Massimo Iorizzo, Gianluca Albanese, Bruno Testa *, Mario Ianiro, Francesco Letizia, Mariantonietta Succi, Patrizio Tremonte, Mariasilvia D'Andrea, Nicolaia Iaffaldano and Raffaele Coppola**

Department of Agriculture, Environmental and Food Sciences, University of Molise, Via De Sanctis, 86100 Campobasso, Italy; iorizzo@unimol.it (M.I.); g.albanese@studenti.unimol.it (G.A.); m.ianiro@studenti.unimol.it (M.I.); f.letizia@studenti.unimol.it (F.L.); succi@unimol.it (M.S.); tremonte@unimol.it (P.T.); dandrea@unimol.it (M.D.); nicolaia@unimol.it (N.I.); coppola@unimol.it (R.C.)
* Correspondence: bruno.testa@unimol.it

**Abstract:** Knowledge of the composition of the gut microbiota in freshwater fish living in their natural habitat has taxonomic and ecological importance. Few reports have been produced on the composition of the gut microbiota and on the presence of LAB in the intestines of freshwater fish that inhabit river environments. In this study, we investigated the LAB community that was present in the gastrointestinal tract (GIT) of Mediterranean trout (*Salmo macrostigma*) that colonized the Biferno and Volturno rivers of the Molise region (Italy). The partial 16S rRNA gene sequences of these strains were determined for the species-level taxonomic placement. The phylogenetic analysis revealed that the isolated LABs belonged to seven genera (*Carnobacterium*, *Enterococcus*, *Lactobacillus*, *Lactiplantibacillus*, *Vagococcus*, *Lactococcus*, and *Weissella*). The study of the enzymatic activities showed that these LABs could contribute to the breakdown of polysaccharides, proteins, and lipids. In future studies, a greater understanding of how the LABs act against pathogens and trigger the fish immune response may provide practical means to engineer the indigenous fish microbiome and enhance disease control and fish health.

**Keywords:** lactic acid bacteria; gut microbiota; Mediterranean trout; river environment

**Citation:** Iorizzo, M.; Albanese, G.; Testa, B.; Ianiro, M.; Letizia, F.; Succi, M.; Tremonte, P.; D'Andrea, M.; Iaffaldano, N.; Coppola, R. Presence of Lactic Acid Bacteria in the Intestinal Tract of the Mediterranean Trout (*Salmo macrostigma*) in Its Natural Environment. *Life* **2021**, *11*, 667. https://doi.org/10.3390/life11070667

Academic Editor: Peter Deines

Received: 27 April 2021
Accepted: 5 July 2021
Published: 7 July 2021

**Publisher's Note:** MDPI stays neutral with regard to jurisdictional claims in published maps and institutional affiliations.

**Copyright:** © 2021 by the authors. Licensee MDPI, Basel, Switzerland. This article is an open access article distributed under the terms and conditions of the Creative Commons Attribution (CC BY) license (http://creativecommons.org/licenses/by/4.0/).

**Supplementary Materials:**

**Table S1.** List of the isolates LABs with corresponding GenBanK (NCBI) accession numbers and the taxonomic references.

| ID Bacterial Strains | Taxonomic Reference | Accession Number (GenBank) |
|---|---|---|
| L1 | *Carnobacterium maltaromaticum* | MZ452146 |
| L2 | *Carnobacterium maltaromaticum* | MZ452147 |
| L3 | *Carnobacterium maltaromaticum* | MZ452148 |
| L5 | *Carnobacterium maltaromaticum* | MZ452149 |
| L9 | *Carnobacterium maltaromaticum* | MZ452150 |
| L11 | *Carnobacterium maltaromaticum* | MZ452151 |
| L13 | *Carnobacterium maltaromaticum* | MZ452152 |
| L14 | *Carnobacterium maltaromaticum* | MZ452153 |
| L18 | *Carnobacterium maltaromaticum* | MZ450132 |
| L20 | *Carnobacterium maltaromaticum* | MZ450133 |
| B5 | *Carnobacterium maltaromaticum* | MZ450134 |
| B7 | *Carnobacterium maltaromaticum* | MZ452154 |
| M21C | *Carnobacterium maltaromaticum* | MZ452155 |
| M21CR | *Carnobacterium maltaromaticum* | MZ452156 |
| 2CR | *Carnobacterium maltaromaticum* | MZ452157 |
| 2ACR | *Carnobacterium maltaromaticum* | MZ452158 |
| 11V | *Carnobacterium maltaromaticum* | MZ452159 |
| 12V | *Carnobacterium maltaromaticum* | MZ452160 |
| 18V | *Carnobacterium maltaromaticum* | MZ452161 |
| 25V | *Carnobacterium maltaromaticum* | MZ450135 |
| 42V | *Carnobacterium maltaromaticum* | MZ452162 |
| 43V | *Carnobacterium maltaromaticum* | MZ452163 |
| 45V | *Carnobacterium maltaromaticum* | MZ452164 |
| 46V | *Carnobacterium maltaromaticum* | MZ452165 |
| 1T | *Carnobacterium maltaromaticum* | MZ452166 |
| 2T | *Carnobacterium maltaromaticum* | MZ452167 |
| 3T | *Carnobacterium maltaromaticum* | MZ452168 |
| 4T | *Carnobacterium maltaromaticum* | MZ452169 |
| B2 | *Lactiplantibacillus plantarum* | MZ452091 |
| 23V | *Lactiplantibacillus plantarum* | MZ452092 |
| 33V | *Lactiplantibacillus plantarum* | MZ452093 |
| 36V | *Lactiplantibacillus plantarum* | MZ452094 |
| 37V | *Lactiplantibacillus plantarum* | MZ452095 |
| 38V | *Lactiplantibacillus plantarum* | MZ452096 |
| 63V | *Lactiplantibacillus plantarum* | MZ452097 |
| 64V | *Lactiplantibacillus plantarum* | MZ452098 |
| 65V | *Lactiplantibacillus plantarum* | MZ452099 |
| 66V | *Lactiplantibacillus plantarum* | MZ452100 |
| 67V | *Lactiplantibacillus plantarum* | MZ452101 |
| 68V | *Lactiplantibacillus plantarum* | MZ452102 |
| 73V | *Lactiplantibacillus plantarum* | MZ452103 |
| 6T | *Lactiplantibacillus plantarum* | MZ452104 |
| 3V | *Lactobacillus acidophilus* | MZ452105 |
| L8 | *Lactiplantibacillus pentosus* | MZ452106 |
| L10 | *Lactococcus lactis* | MZ452076 |

| | | |
|---|---|---|
| L12 | *Lactococcus lactis* | MZ452077 |
| L14B | *Lactococcus lactis* | MZ452078 |
| L19 | *Lactococcus lactis* | MZ452079 |
| B6 | *Lactococcus lactis* | MZ452080 |
| B34 | *Lactococcus lactis* | MZ452081 |
| L4 | *Enterococcus faecalis* | MZ452069 |
| 2V | *Enterococcus faecalis* | MZ452070 |
| 4V | *Enterococcus faecalis* | MZ452071 |
| 7V | *Enterococcus faecalis* | MZ452072 |
| 31V | *Enterococcus faecalis* | MZ452073 |
| 32V | *Enterococcus faecalis* | MZ452074 |
| 34V | *Enterococcus faecalis* | MZ452075 |
| 15V | *Vagococcus fluvialis* | MZ451958 |
| 39V | *Weissella paramesenteroides* | MZ451959 |
| 57V | *Weissella paramesenteroides* | MZ451960 |
| 61V | *Weissella paramesenteroides* | MZ451961 |
